# Almost complete solution for the NP-hard separability problem of Bell diagonal qutrits

**DOI:** 10.1038/s41598-022-16225-z

**Published:** 2022-07-21

**Authors:** Christopher Popp, Beatrix C. Hiesmayr

**Affiliations:** Faculty of Physics, University of Vienna, Währingerstrasse 17, 1090 Vienna, Austria

**Keywords:** Quantum physics, Quantum information, Quantum mechanics

## Abstract

With a probability of success of 95% we solve the separability problem for Bell diagonal qutrit states with positive partial transposition (PPT). The separability problem, i.e. distinguishing separable and entangled states, generally lacks an efficient solution due to the existence of bound entangled states. In contrast to free entangled states that can be used for entanglement distillation via local operations and classical communication, these states cannot be detected by the Peres–Horodecki criterion or PPT criterion. We analyze a large family of bipartite qutrit states that can be separable, free entangled or bound entangled. Leveraging a geometrical representation of these states in Euclidean space, novel methods are presented that allow the classification of separable and bound entangled Bell diagonal states in an efficient way. Moreover, the classification allows the precise determination of relative volumes of the classes of separable, free and bound entangled states. In detail, out of all Bell diagonal PPT states $81.0\% \pm 0.1\%$ are determined to be separable while $13.9 \pm 0.1\%$ are bound entangled and only $5.1 \pm 0.1\%$ remain unclassified. Moreover, our applied criteria are compared for their effectiveness and relation as detectors of bound entanglement, which reveals that not a single criterion is capable to detect all bound entangled states.

## Introduction

Quantum information theory can be understood as the study of processing tasks that can be accomplished using quantum mechanical systems^1^. Quantum technology uses quantum phenomena for improved or new technological applications. Prominent fields include quantum computing, quantum communication, quantum simulation, quantum metrology or quantum cryptography. Most of existing experimental and theoretic applications of quantum technology are based on two-level quantum systems, called “qubits”($d=2$). Recently however, interest in higher dimensional systems, i.e. “qutrits” ($d=3$) or “qudits” (general *d*) is growing^2,3^. Although experimentally more challenging, qudits are expected to have certain advantages and show new phenomena. This work wants to explore one of these phenomena.

Entanglement is one of the main resources to realize information processing with better performance than any classical system and has been shown to exist in many physical systems and is intensively explored for potential applications ranging from quantum teleportation to the detection of cancer in human beings^4–7^. Despite its theoretical and practical relevance, there is no general method to determine whether a bipartite quantum state is entangled or not, in which case the state is separable. This problem is strongly connected to a special form of entanglement called “bound entanglement” for which a first example has been found by the Horodecki family in 1998^8^ and has been studied since then, e.g. Refs.^9–19^, and has been experimentally verified with photons entangled in the orbital momentum degrees of freedom^20^. Often, several weakly entangled states can be transformed via local operations and classical communication (LOCC) to a smaller set of strongly entangled states. This process is called entanglement distillation^8^ and is of high relevance for practical applications that require strong distillable or “free” entanglement as a resource. For bound entangled states, this distillation is not possible. However, they can be generated by mixing several free entangled states, binding or preventing their entanglement to be further used in applications that require distillation, thus motivating the name. In order to avoid operations that would bind the resource of entanglement in bound entangled states through this irreversible mixing process, it is important to have information about the structure of bound entangled states. While free entanglement can be efficiently detected by the the Peres–Horodecki criterion (also known as PPT criterion)^21,22^ in a bipartite system, the detection of bound entanglement has been proved to be NP-hard for bipartite systems and thus intractable for higher dimensions^23^. Up to now, there is no complete understanding about why this special form of entanglement exists, what its existence implies for nature or what its exact capabilities with respect to quantum information theoretic tasks are. Moreover, there is no general method known to construct bound entangled states.

In this paper we investigate mixtures of maximally entangled, bipartite, $3 \times 3$-dimensional (“Bell”)-states. One significant subset of this class of Bell diagonal states has been shown^20,24–26^ to be especially useful to analyze (bound) entanglement due to a representation, which allows a precise geometrical interpretation of the system. Depending on the mixing probabilities of pure maximally entangled states, the mixed state can be separable, bound entangled or free entangled. Although geometric properties allow analytical insight in the structure of entanglement^24^, the problem of entanglement classification, especially the detection of bound entanglement, remains a hard problem. Several methods to detect bound entangled states exist^13–19^, but these methods based on specifically constructed non-decomposable entanglement witnesses or other constructions are strongly limited in the number of states they can detect. Zyczkowski numerically analyzed^27,28^ general higher dimensional systems with respect to the separability problem and entanglement classification and determined approximate relative volumes of separable and bound entangled states for different dimensions. Among other results it was shown that the relative volume of separable and bound entangled states decreases exponentially with the dimension of the system. Recently, Hiesmayr analyzed mixtures of Bell states for $d=3$ to determine relative volumes of separable and (bound) entangled states and to detect relevant substructures in the set of bound entangled states^29^.

This work intends to set up a numeric framework that allows the efficient and precise determination of the entanglement class of Bell diagonal states in general dimension and to apply it to the case $d=3$. An alternative, more precise and efficient way to generate and analyze states in order to estimate the relative volumes of entanglement classes is presented. New methods are developed to efficiently solve the separability problem in the considered system for almost all states. Finally, the methods are compared in their effectiveness for the detection of bound entanglement.

The paper is organized as follows: First, we define the systems to be analyzed and several subsets of relevance for the investigation. Then, a method to efficiently generate states of that system based on random sampling is presented and shown to have significant advantages compared to the methods used in previous related analyses^29^. We show how symmetries of the system can be numerically generated and leveraged for entanglement classification. We then give a short overview over implemented and used methods for the detection of (bound) entangled and separable states, including a new numerical sufficient criterion for separability. Finally, these methods are applied to the system for $d=3$ and some relevant subset, allowing to efficiently determine the entanglement class of 95% of all PPT states.

## Methods

In this paper we analyze mixtures of maximally entangled orthonormal Bell states $\left| {\Omega _{k,l}}\right\rangle \in \mathscr {H} = \mathscr {H}_1 \otimes \mathscr {H}_2$ for $k, l = 0, 1, \cdots , (d-1)$ in the Hilbert space $\mathscr {H}$ of a bipartite system containing two qudits of dimension *d*. Suppose Alice and Bob share a bipartite maximally entangled state $\left| \Omega _{00}\right\rangle \equiv \frac{1}{\sqrt{d}} \sum _{i = 0}^{d-1} \left| ii\right\rangle$. A basis of orthonormal Bell states $\left| {\Omega _{k,l}}\right\rangle$ can be generated by applying the Weyl operators^30^ $W_{k,l} \equiv \sum _{j=0}^{d-1}w^{j \cdot k} \left| j\right\rangle \left\langle j+l \pmod d\right| ,~w = e^{\frac{2 \pi i}{d}},$ to one of the subsystems, without loss of generality Alice’s qudit: $\left| {\Omega _{k,l}}\right\rangle {\equiv }{W}_{{k,l}} {\otimes }{ \mathbbm {1}}_{d} \left| \Omega _{00}\right\rangle$. The density matrices of these pure basis states are then the “Bell projectors” $P_{kl} \equiv \left| {\Omega _{k,l}}\right\rangle \left\langle {\Omega _{k,l}}\right|$. Mixtures of these states define the system of interest $\mathscr {M}_d$ in this work:

1 $$\begin{aligned} \mathscr {M}_d \equiv \left \lbrace \rho = \sum _{k,l = 0}^{d-1}c_{k,l} P_{k,l}~ |~ \sum _{k,l = 0}^{d-1}c_{k,l} = 1, c_{k,l} \ge 0 \right \rbrace. \end{aligned}$$

$\mathscr {M}_d$ has also been named “magic simplex”^24–26^, referring to the “magic Bell basis” introduced by Wootters and Hill^31^ and the fact that it can be represented as simplex in real space, identifying the mixing probabilities $c_{k,l}$ as coordinates with the Bell projectors $P_{k,l}$ lying at the vertices of the simplex. A special property of this set is that the reduced states, i.e. the partial trace with respect to one of the subsystems, of all states in $\mathscr {M}_d$ are maximally mixed. They are therefore said to be “locally maximally mixed” states. Note that for $d>2$, $\mathscr {M}_d$ does not contain all locally maximally mixed states^25^.

Depending on the focus of the analysis, several subsets and families of states are of interest and have been discussed in literature^26,32^. For the entanglement analysis, the following families are especially relevant:

### Enclosure polytope

The enclosure polytope is defined as

2 $$\begin{aligned} \mathscr {E}_d \equiv \lbrace \rho = \sum _{k,l = 0}^{d-1}c_{k,l} P_{k,l}~ |~ \sum _{k,l = 0}^{d-1}c_{k,l} = 1, c_{k,l} \in [0, \frac{1}{d}] \rbrace . \end{aligned}$$

It was shown^24^ that all states that lie outside of the enclosure polytope are necessarily entangled. Since they can be detected by the PPT or Peres-Horodecki criterion, they can be distilled by local operation and classical communication (LOCC), or equivalently, are free and not bound entangled^8^.

### Kernel polytope

The kernel polytope is another geometric object that allows to determine the entanglement class of a given state represented by its coordinates in $\mathscr {M}_d$. It is defined as convex mixture of certain separable states, named “line states” $\rho _{\alpha }$^24^:

3 $$\begin{aligned} \mathscr {K}_d \equiv \lbrace \rho = \sum _\alpha \lambda _{\alpha } \rho _{\alpha }~ |~ \lambda _\alpha \ge 0, \sum _\alpha \lambda _\alpha = 1 \rbrace . \end{aligned}$$

The line states $\rho _\alpha$ are related to cyclic subgroups (or more general sublattices in higher dimensions) of the linear ring structure induced by the Weyl operators. As those are separable states, each state in the $\mathscr {K}_d$ is separable by construction.

### Family A

Family A is defined by a mixture of three Bell states and the maximally mixed state $\rho _{mm} = \frac{1}{d^2} \sum _{k,l = 0}^{d-1}P_{k,l}= \frac{1}{d^2} \sum _{i,j = 0}^{d-1} \left| ij\right\rangle \left\langle ij\right|$ for $d=3$. The three Bell states are required to be on a phase space line^24^, i.e. one Bell state is chosen and the other two are generated by application of one chosen Weyl transformation. Choosing without loss of generality the Weyl transformation to be ${W}_{{0,1}}{\otimes }{\mathbbm {1}}_{{d}}$, the states of Family A can explicitly be written as

4 $$\begin{aligned} \mathscr {F}_A \equiv \lbrace \rho _A = \alpha P_{00} + \beta P_{01} + \gamma P_{02} + (1-\alpha -\beta - \gamma ) \rho _{mm}~|~ \rho _A \in M_3 \rbrace . \end{aligned}$$

Leveraging the high symmetry of such states, an optimal entanglement witness was found^24^ and later related to the quasi-pure approximation of the concurrence criterion^32^. It is therefore possible to determine the entanglement class of all states in this family.

### Numerical generation of states

The representation of the system as $(d^2-1)$-dimensional simplex with the $d^2$ Bell states as vertices and the probabilities as “baryocentric coordinates” allows the efficient geometric representation of states as points in the simplex. In this representation, states for the whole simplex, subsets of finite volume or hyper-planes can be equivalently described and generated as points in $d^2$-dimensional Euclidean space. This can be achieved by, e.g., random sampling according to a certain distribution or by some deterministic discretization procedure. In the following, two ways that allow the estimation of relative volumes of separable, bound and free entangled states are shortly described.

#### Uniform random sampling of states

The linear, real (sub-)spaces presented above allow uniform sampling of points within and therefore uniformly distributed states can be generated in $\mathscr {M}_d$, $\mathscr {E}_d$ or $\mathscr {F}_A$. Classification of these states allows a probabilistic estimation of relative volumes of the entanglement classes by using the relative number/frequency of classified states as estimator. As the number of required states for a valid estimation only depends of the relative volumes of the classes and not on the dimension, this method of state creation is favorable for volume estimation in higher dimensions. Another advantage of random sampling compared to some deterministic procedure is that the expected relative number of states in a given class only depends on the volume and not on the (unknown) specific geometric shape of the set of states within that class.

**Figure 1:**
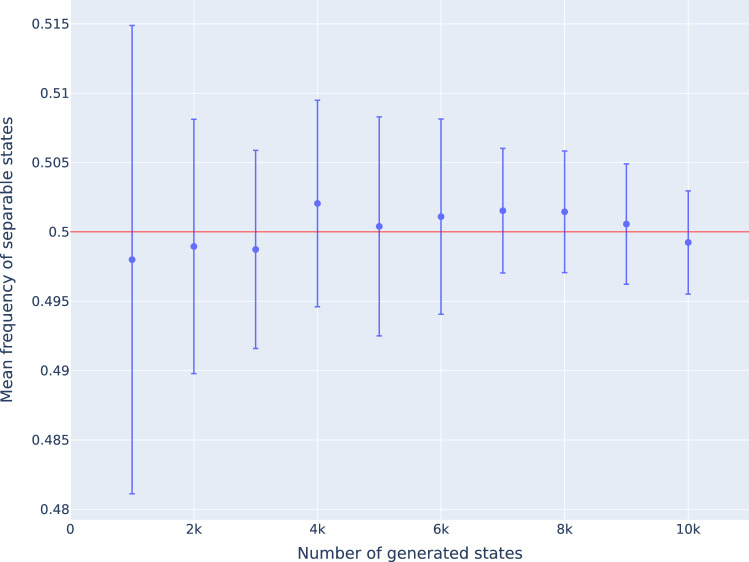
Relative frequencies of separable states in $M_2$. For each sample size, 10 sets of randomly generated states are generated. The figure shows the empirical mean and standard deviation for the frequencies of separable states.

Let us demonstrate the validity of this method to estimate the relative volumes of entanglement classes for bipartite qubits (i.e. $d=2$). In this case, the separability problem can be solved analytically. It has been shown that for $d=2$, all entangled states are free entangled and can be detected with the PPT criterion^21,22^ and therefore $\mathscr {M}_2$ contains no bound entanglement. It is also known that the kernel polytope $\mathscr {K}_2$ contains all separable states and that the relative volumes of both classes in $\mathscr {M}_2$ are exactly 50%. Figure 1 shows the relative frequency of separable states for uniform samples in $\mathscr {M}_2$. For different sample sizes, the empirical mean and standard deviation of 10 runs are presented. One observes that the relative frequency converges with growing number of states to the known equal sized relative volumes of 0.5 with increasing precision. Given a random sample of size *N*, the number of states within a certain class of relative volume *p* should be distributed according to the binomial distribution. The expected number of states in that class is then $N \cdot p$ with standard deviation of $\sqrt{N p (1-p)}$. In case of $\mathscr {M}_2$, the probability that a generated state is PPT and in this special case also separable is $p=0.5$.

**Table 1:** Number of PPT states in sample sets of size 10,000.

Sample set	#PPT states
1	4869
2	5021
3	4901
4	5019
5	4982
6	4996
7	5014
8	5022
9	4969
10	5079

Table shows ten sample sets containing 10,000 states each and the number of PPT states within. The empirical mean is 4987.2 with empirical standard deviation of 61.7. These results are in agreement with the expected mean of 5000 and standard deviation of 50 according to the binomial distribution. For the exemplary analysis below we use a sample size of 10,000 states. The determined empirical values imply that the obtained numbers for the size of relative volumes have an statistical error in the magnitude of $10^{-3} = 0.1\%$.

#### States on an equidistant lattice

**Figure 2:**
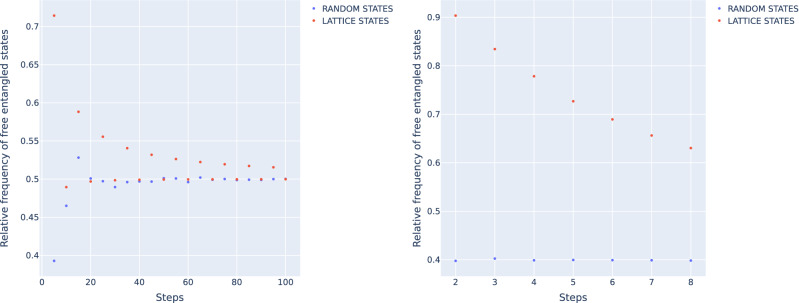
Relative frequency of free entangled states generated on a lattice (red) and by random sampling (blue) in dependence of the number of steps for $\mathscr {M}_2$ for $d=2$ (left) and $\mathscr {E}_3$ for $d=3$ (right).

One way to discretize the space of interest is using a constant increment in all dimensions. Given $k \le (d^2-1)$ independent parameters, a certain range *R* of values they can assume and a number of steps *s* to divide the range in, one obtains $(s+1)^k$ lattice points. Generating states on a fixed lattice offers the advantage that these states have many symmetries, allowing to reduce the number of states to explicitly classify. On the other hand, generating the full lattice is quickly too computationally expensive with growing dimension, due to the exponential dependency of the number of lattice points given a certain number of steps. This makes this discretization computationally inefficient in higher dimensions, because the step size needs to be small enough to capture the entanglement structure. Another disadvantage of this set of states is that they are only approximately uniformly distributed for small increments. One example for that is the over-representation of states in certain areas depending on the chosen discretization. As a consequence, classifying the states and counting the relative occurrences as estimator for the relative volumes of the entanglement classes is unbiased only in the limit of large *s*. Figure 2 compares the relative frequency of states that are entangled according to the PPT criterion for random samples and states on a fixed lattice. For $d=2$, the frequencies are relative to the whole simplex $\mathscr {M}_2$, while for $d=3$ the enclosure polytope $\mathscr {E}_3$ is used. The ranges of the coordinates are divided in a certain amount of steps to generate the states on a lattice. For comparison, an equal number of random states is generated and analyzed. In Fig. 2 (left) one observes that the randomly generated states quickly converge to the known relative volume of 0.5. The lattice states show a strong dependence on the number of steps. While for an even number of steps the relative frequency approaches the volume from below, for odd numbers the convergence is slower and from above. Figure 2 (right) shows that already for $d=3$, the number of lattice states that can be generated by standard computational means is not high enough to avoid a significant bias compared to the quickly converging randomly generated states.

### Generation of symmetry transformations conserving the entanglement property

The phase space structure of $\mathscr {M}_d$ implies a set of ”entanglement-conserving” symmetry transformations related to point transformations and translations in the discrete phase space induced by Weyl operators^24,25^. They act as permutations of the generating Bell projectors, or equivalently, as permutations of the coordinates in $\mathscr {M}_d$ and are generated by elementary transformations, i.e. generators of the involved symmetry group. These generators can be identified with translation, momentum inversion, quarter rotation and vertical shear in the induced discrete phase space (see Supplementary Appendix A3). It was shown that these symmetries conserve entanglement in $\mathscr {M}_d$. A simple argument (see A1 in the Supplementary Appendix) also shows that the entanglement class is conserved, meaning that the classification of free, bound and separable states is not changed by applying any element of this group. The generators and their actions can be represented as specific permutations, allowing to generate all elements by succeeding application of several symmetry transformations. Since the phase space contains a finite number of elements, also the number of permutations is finite. This allows to determine all symmetries generated by the above generators in arbitrary dimension. The number of symmetries that can be generated by those generators grows quickly with the dimension of the subsystems. For $d=2$, 24 such symmetries exist, while for $d=3$ already 432 symmetries can be found. Leveraging these symmetries is crucial to the analysis of entanglement in $\mathscr {M}_d$, significantly improving the differentiation of bound entangled from separable states.

### Sufficient criteria for entanglement

The problem to decide whether a given state is separable or entangled is generally a NP-hard problem^23^. No general solution by polynomial in time algorithms is known and it is often denoted the “separability problem”. Likewise, there is no efficient method to solve the separability problem for the mixtures of maximally entangled states $\mathscr {M}_d$ in general dimension *d*. However, several sufficient criteria to detect entanglement are known and can be used for entanglement classification. The most effective ones for states in $\mathscr {M}_d$ are shortly stated below and enumerated by E1, E2,....

#### E1: PPT criterion

The “Positive Partial Transpose (PPT)” criterion^21^, also named “Peres–Horodecki” criterion states that if the partial transpose of a bipartite state has at least one negative eigenvalue (in this case the state is said to be “NPT”), it is entangled. The partial transpose $\Gamma$ acts on the basis states of a bipartite state as $(\left| i\right\rangle \left\langle j\right| \otimes \left| k\right\rangle \left\langle l\right| )^{\Gamma } \equiv \left| i\right\rangle \left\langle j\right| \otimes \left| l\right\rangle \left\langle k\right|$. It can be calculated efficiently, but for $d \ge 3$ it is only sufficient, not necessary, for entanglement. In this case, also states that have a positive partial transpose can be entangled. Only entangled NPT-states can be used for entanglement distillation are therefore denoted as free entangled.

#### E2: realignment criterion

The realignment criterion^33^ is structurally similar to the PPT criterion. The realignment operation *R* acts as $(\left| i\right\rangle \left\langle j\right| \otimes \left| k\right\rangle \left\langle l\right| )_R \equiv \left| i\right\rangle \left\langle k\right| \otimes \left| j\right\rangle \left\langle l\right|$. The criterion states that if the sum of singular values of the realigned state $\sigma _R$ are larger than 1, i.e. if $\hbox {tr}\sqrt{\sigma _R^{\dagger } \sigma _R} > 1$, then $\sigma$ is entangled. Like the PPT criterion, it can also be computed efficiently, but is only sufficient for entanglement. It is neither stronger or weaker that the PPT criterion, so it can detect PPT entangled states, but generally does not detect all NPT-states.

#### E3: quasi-pure concurrence criterion

The quasi-pure approximation $C_{qp}$ of the concurrence^34^ provides another sufficient entanglement criterion. States for which the concurrence is positive are entangled, but for $d>2$ the concurrence allows only numerical estimates for mixed states. The quasi-pure approximation^32^ provides an easy to compute lower bound for the concurrence and can therefore be used for entanglement detection. Considering states of $\mathscr {M}_d$, an explicit form for the approximation can be derived. A state $\rho = \sum _{k,l = 0}^{d-1}c_{k,l}P_{k,l} \in \mathscr {M}_d$ is entangled according to the quasi-pure concurrence criterion if $C_{qp}(\rho ) = \max (0, S_{nm} - \sum _{(k,l) \ne (n,m)} S_{k,l})>0$ where (*n*, *m*) is a multi-index of the coordinate of the largest mixing probability $\lbrace c_{k,l} \rbrace$ and $S_{k,l}$ are explicitly given by^32^:

5 $$\begin{aligned} S_{k,l} = \sqrt{ \frac{d}{2(d-1)} c_{k,l} [(1-\frac{2}{d}) c_{n,m} \delta _{k,n} \delta _{l,m} + \frac{1}{d^2} c_{(2n-k)mod~d,(2m-l)mod~d}] }. \end{aligned}$$

#### E4: MUB criterion

In a Hilbert space of dimension *d*, a set of orthonormal bases $\lbrace B_k \rbrace$ and $B_k = \lbrace \left| i_k\right\rangle ~|~ i = 0,\ldots , (d-1) \rbrace$ is called “mutually unbiased bases (MUB)” if $\forall k \ne l$:

6 $$\begin{aligned} |\left\langle i_k\vert j_l\right\rangle |^2 = \frac{1}{d} ~~~ \forall i,j = 0, \ldots , (d-1). \end{aligned}$$

Given *m* MUBs, it was shown^20,35^ that the sum of all “mutual predictabilities“ $I_m$ is bounded from above for separable states $\rho _s$:

7 $$\begin{aligned} I_m(\rho _s) = \sum _{k=1}^m C_k = \sum _{k=1}^m \sum _{i=0}^{d-1} \left\langle i_k\right| \otimes \left\langle i_k\right| \rho _s \left| i_k\right\rangle \otimes \left| i_k\right\rangle \le 1 + \frac{m-1}{d}. \end{aligned}$$

At most $d+1$ MUBs exist^36,37^ in which case $I_{d+1}(\rho _s) \le 2$ for all separable states $\rho _s$. Conversely, if an unclassified state exceeds this upper bound, it is entangled. In order to detect bound entanglement in $d=3$, the $C_k$ can be modified to the following form:

8 $$\begin{aligned} C_1= & {} \sum _{i=0}^{2} \left\langle i_1\right| \otimes \left\langle (i_1+2\pmod 3)^*\right| \rho _s \left| i_1\right\rangle \otimes \left| i_1+2\pmod 3^*\right\rangle , \end{aligned}$$

9 $$\begin{aligned} C_k= & {} \sum _{i=0}^{2} \left\langle i_k\right| \otimes \left\langle i_k^*\right| \rho _s \left| i_k\right\rangle \otimes \left| i_k^*\right\rangle ,~ k=2,3,4. \end{aligned}$$

Here, $i_k^*$ denotes the complex conjugate vector element. In this form, like the realignment and quasi-pure concurrence criteria, the MUB criterion allows the detection of PPT entangled states, which was also experimentally demonstrated for entangled photons^20^.

#### E5: numerically generated entanglement witnesses

Leveraging the fact that separable states form a convex set, entanglement witnesses^38^ (“EWs”) are an important tool to detect entangled states. An EW *W* is an observable which implies an upper bound *U* and, as recently shown^39^, also a lower, mostly nontrivial, bound *L* ($U,L \in {\mathbb {R}}$), for separable states $\rho _s$:

10 $$\begin{aligned} L \le \hbox {tr}[\rho _s W] \le U. \end{aligned}$$

I﻿f﻿ a state $\rho$ suffices $\hbox {tr}[\rho W] \notin [L,U]$ for some *W*, then it is entangled and said to be “detected by *W*”. Originally EWs were introduced considering the upper bound $U=0$ only. It has been shown^24^ that for each entangled state $\rho \in \mathscr {M}_d$ there exists an EW $W_\rho$ such that $W_\rho$ detects $\rho$. In this sense, EWs are universal, because each entangled state can be detected by an, unfortunately generally unknown, EW. If $\rho \in \mathscr {M}_d$, it suffices to consider EWs of the form $W = \sum _{k,l = 0}^{d-1}\kappa _{k,l} P_{k,l}$ with $\kappa _{k,l} \in [-1,1]$ to detect all entangled states^24^. In this case $\rho = \sum _{k,l = 0}^{d-1}c_{k,l}P_{k,l}\in \mathscr {M}_d$ and we have $\hbox {tr}[\rho W]= \sum _{k,l = 0}^{d-1}c_{k,l} \kappa _{k,l} \equiv c \cdot \kappa$ where the dot indicates the standard scalar product of the $d^2$-dimensional vectors *c* and $\kappa$ collecting the coefficients $c_{k,l}$ and $\kappa _{k,l}$. Thus, in the geometric representation of $\mathscr {M}_d$, any EW defines two $(d^2-1)$-dimensional hyper-planes via $c_L \cdot \kappa = L$ and $c_U \cdot \kappa = U$ and corresponding halfspaces. Any state that is represented by a point outside of the intersection of these halfspaces is detected as entangled. The difficulty of using EWs lies of course in determining the bounds *L* and *U* for the set of separable states. An efficient parameterization of unitaries^40^ and numerical optimization^41^ is used to determine the bounds for our case (see Supplementary Appendix 2 for more details).

### Sufficient criteria for separability

Since the presented criteria for entanglement are only sufficient and generally not necessary, it is highly desirable to develop methods to directly classify separable states in as well. Few analytical sufficient criteria for separability exist, but the geometric characterization and properties of $\mathscr {M}_d$ allow the development of an effective procedure to identify states of $SEP \cap \mathscr {M}_d$. Here we present a new method based on the extension of the convex hull of known separable states (S1) and a criterion with close relation to the states of $\mathscr {M}_d$ (S2).

#### S1: extended kernel criterion

Given a finite set of known separable states and their representation in $\mathscr {M}_d$, a convex polytope can be constructed by building the convex hull of those vertices. All states of $\mathscr {M}_d$ with coordinates within this polytope are separable as well. The problem to decide whether a given Bell diagonal state is a convex combination of known existing separable states is then equivalent to the standard problem of linear programming to decide whether a given point lies within a convex polytope. Several numerical implementations to solve that problem exist, e.g. Ref.^42^, which was used for this work. Obviously, in order to check an unknown state $\rho \in \mathscr {M}_d$ for separability, a set of known separable states as vertices to build the convex hull are required. Since the effectivity of the check depends on the volume of the spanned polytope, it is advantageous to use vertices that cover uniformly distributed spatial angles and are as close to the boundary of the convex set of separable states as possible. The line states $\rho _\alpha$ that build the kernel polytope $\mathscr {K}_d$ meet those requirements, as they are known to be on the surface of *SEP*^24^. However, more separable states/vertices are needed to cover approximately all separable states in $\mathscr {M}_d$. For the results presented in this work, additional separable states were generated and multiplied by using the entanglement-class-conserving symmetries to generate more vertices to extend the separable kernel.

#### S2: Weyl/spin representation criterion

The Weyl operators $W_{k,l}$ satisfy useful relations to simplify required calculations for states of $\mathscr {M}_d$. Two of those are:

11 $$\begin{aligned} W_{k_1,k_2}W_{l_1,l_2}= & {} w^{k_2 l_1}W_{k_1+l_1, k_2+l_2}, \end{aligned}$$

12 $$\begin{aligned} W_{k_1,k_2}^\dagger= & {} w^{k_1 k_2}W_{-k_1, -k_2} = W_{k_1, k_2}^{-1}. \end{aligned}$$

It follows that the Weyl operators form an orthogonal family of unitary matrices with respect to the trace norm $(A|B) \equiv \hbox {tr}[A^\dagger B]$ and as such is a basis for the space of $d \times d$ matrices. Any density matrix $\sigma$ can then be represented as $\sigma = \frac{1}{d} \sum _{k,l = 0}^{d-1}s_{k,l} W_{k,l}$. The coefficients of this Weyl representations are $s_{k,l} = \hbox {tr}[W_{k,l}^\dagger \sigma ]$. Accordingly, $W_{\mu , \nu } \equiv W_{\mu _1, \nu _1} \otimes W_{\mu _2, \nu _2}$ can be used to represent a bipartite state $\rho$ with coefficients $s_{\mu , \nu }$. A sufficient criterion for separability was derived^43^ that is named “Weyl” or “Spin representation criterion” here: If $\sum _{\mu , \nu } |s_{\mu , \nu }| \le 2$, where $|s_{\mu , \nu }|$ are the coefficients of the Weyl representation of the bipartite state $\rho$, then $\rho$ is separable.

### Symmetry analysis

Leveraging the rich symmetries of $\mathscr {M}_d$ is another crucial factor to detect both entanglement and separability with the presented methods. The generation of all elements of this symmetry group greatly improves the detection capabilities of available detectors. Given an unknown state, all symmetric states can generated by application of the according transformation for all generated symmetries. This set is then analyzed with respect to the available criteria. If entanglement class is determined for one state, all states of the set of symmetric states are known to be of the same class as well, due to the entanglement class conserving property of the symmetries.

## Results

We determine the share of separable, bound and free entangled states with high accuracy using the methods presented above. After summarizing arguments for the generation of states via random sampling, two exemplary analyses are presented. First, states of the subset $\mathscr {F}_A$ (Eq. (4)) are analyzed before the main results of the entanglement classification for the enclosure polytope $\mathscr {E}_3$ (Eq. (2)) for the $d=3$ are presented. The analysis of $\mathscr {F}_A$ serves as validation and comparison of applied methods, because here the borders between separable, bound entangled and free entangled states are analytically known^25,32^. Since we know that all states outside of the enclosure polytope $\mathscr {E}_d$ are free entangled, the analysis of $\mathscr {E}_3$ suffices to know the entanglement classification of the whole simplex $\mathscr {M}_3$. Entanglement and separability criteria are compared for their effectiveness and their relations are discussed. The states are classified according to the labels “SEP” (separable states), “BOUND” (bound/PPT entangled states), “FREE” (free/NPT entangled states) and “PPT-UNKNOWN” (PPT states that could not be shown to be separable or bound entangled). For that purpose, more than 16,000 entanglement witnesses have been generated numerically. Additionally, the kernel of separable states has been extended by new separable states leveraging the generated symmetries to generate new vertex states for the convex hull. The symmetries have also been used for all methods to further increase the detection capabilities (see “Symmetry analysis” section above).

### Generation of states

The generation of uniformly distributed random states offers significant advantages compared to states on a fixed lattice for estimating the relative volumes of entanglement classes in subsets of $\mathscr {M}_d$.

First, the relative frequencies of states in a certain class provides an unbiased estimator for the relative volume of that class if the states are randomly sampled but not if they are generated on a lattice with fixed increment. As shown in Fig. 2 (right), the relative frequencies of randomly generated states converge quickly with increasing sample size, while the convergence of lattice states is slower for $d=3$ so that a significant bias remains for a number of states of the same magnitude.

Second, the expected relative frequencies of random states only depend on the relative volume of the classes and not on their specific geometric shape. A fixed lattice, however, may not distribute its states equally between the classes (according to their volume) as can be observed in Fig. 2 (left). For $d=2$, the relative frequencies depend strongly on the increment of the lattice and show different convergence rates and directions for even and odd number of lattice points in each dimension of $\mathscr {M}_2$. Since the geometric shape of entanglement classes in $\mathscr {M}_d$ is generally unknown for $d \ge 3$, it is not possible to make quantitative statements about the estimation and its error. Here, the uniform generation of random states is advantageous as well, since the probability of finding a random state to be in a given entanglement class is equal to its relative volume. The expected number of states in that class and the standard deviation thereof is then given by the binomial distribution, which was demonstrated for $d=2$ in Table 1. Even if the exact size of the classes (and therefore the distribution) is not known *a priori*, the obtained estimations can be used to approximate the expected deviation of the true volume and the estimation.

Finally, the validity of this method allows the extension for higher dimensions. While already for $d=3$ it is not possible to generate enough states based on a lattice to estimate the relative volumes correctly, the problem will be even larger for $d > 3$ due to the exponential growth of the Hilbert space. For randomly generated states, however, the estimation and its variance depend only on the size of the classes and not on the dimension. This implies that the sample size does not need to be increased for an estimation of the same quality in higher dimensions.

### Entanglement classification for $\mathscr {F}_A$

To analyze Family A, the parameters $\alpha , \beta , \gamma$ of Eq. (4) are uniformly sampled within the range $[-1,1]$ and the corresponding states are checked for positivity. This way, 10,000 uniformly distributed states of $\mathscr {F}_A$ are generated and analyzed.

**Figure 3:**
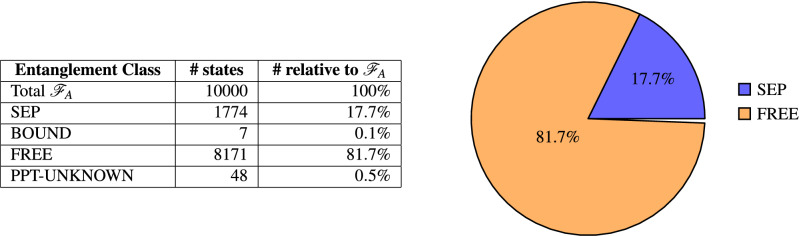
Entanglement classes and their relative volumes in $\mathscr {F}_A$.

Figure 3 shows that more than 99.5% of the states could be classified. The majority of states in $\mathscr {F}_A$ are free entangled (81.7%), 17.7% are separable and only 0.1% of all states are found to be bound entangled. For the remaining 0.5%, the states are known to have a positive partial transpose, but none of the criteria detected separability or bound entanglement.

As mentioned above, the criterion *E*3 was shown to be optimal for this family. This is also reflected in our results in Table 2, showing that only E3 detects bound entangled states. It can therefore be concluded, that the PPT-UNKNOWN states should be separable as well. Concerning separable states, both criteria S1 and S2 detect a significant number of states in SEP. However S1 is clearly stronger than S2, because all states detected by S2 are also detected by S1.

**Table 2:** BOUND and SEP detectors in $\mathscr {F}_A$ and their relative number in class.

Entanglement class	Criterion	#Detected	#Relative to class (%)
SEP	S1	1774	100
SEP	S2	457	25.8
BOUND	E2	0	0
BOUND	E3	7	100
BOUND	E4	0	0
BOUND	E5	0	0

### Entanglement classification for $\mathscr {E}_3$

The main results of this contribution is the determination of the entanglement class for arbitrary mixtures of Bell diagonal states of which $\mathscr {F}_A$ is a small subset. We restrict the analysis to the enclosure polytope $\mathscr {E}_3$ since all other states of $\mathscr {M}_3$ are known to be free entangled according to the PPT criterion.

**Figure 4:**
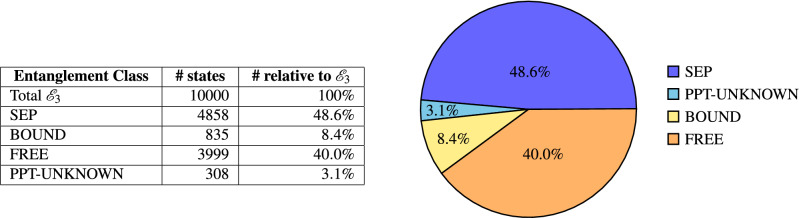
Entanglement classes and their relative volumes in $\mathscr {E}_3$.

For this analysis, 10, 000 uniformly distributed random states are generated and analyzed. The results are summarized in Fig. 4, showing the number of states and the relative volume for each class. Almost half of the states (48.6%) are shown to be separable states via the separability criteria. 48.4% are entangled, of which 40.0% are found to be free entangled according to the PPT criterion as already expected with the analysis of Fig. 2. 8.4% of the states are bound entangled and only 3.1% of the states are known to be PPT-states, but none of the criteria allowed the certain detection of entanglement or separability, so the entanglement class remains unknown. To confirm the classification, we applied the detection criteria for the other classes also to already classified states, which all failed.

These results extend the previous related investigation^29^ in several ways. First, the presented methods allow a more exact estimation of the relative volumes of the entanglement classes in $\mathscr {M}_3$. As argued above, the random generation of states provides an unbiased estimator for the relative volumes. In the cited analysis, the states were generated on a lattice with constant increment, but as shown in Fig. 2 the increment is not small enough to ignore the systematic bias due to the fixed lattice. In the cited analysis, higher values for the relative volumes of FREE and BOUND entangled states are given, while less states are classifies as SEP. Figure 2 indicates that the lattice construction generates a higher density of states in areas in which FREE and BOUND entangled states dominate. Consequently, the generated states are not uniformly distributed, but, as can be easily seen, have a higher density for larger distances to the center of the kernel $\mathscr {K}_3$. This introduces a shift in the relative frequencies of the entanglement classes of generated states, since on average, separable states are closer to the center than entangled states. Second, using the new method S1 and S2 allows the direct detection of separable states beyond the kernel polytope $\mathscr {K}_3$. Together with the creation and consideration of all available symmetries it is possible to significantly reduce the number of PPT-UNKNOWN states.

The main challenge in the classification is of course the differentiation of separable and bound entangled states. For this reason we are strongly interested in the detection capabilities of different criteria for these classes, which are summarized in Table 3.

**Table 3:** BOUND and SEP detectors and their relative number in class.

Entanglement class	Criterion	#Detected	#Relative to class (%)
SEP	S1	4858	100
SEP	S2	76	1.7
BOUND	E2	625	74.9
BOUND	E3	160	19.1
BOUND	E4	113	13.5
BOUND	E5	724	86.6

Although the analytical separability criterion S2 detects some states, the share (1.7% is very small and the criterion S1, leveraging the geometric representation of the system and its symmetries, is clearly stronger. It detects all states for which separability is implied by S2 and many additional ones. Concerning the detection of bound entangled states, two criteria are especially powerful: First the analytical criterion E2, which detects 74.9% of all determined bound entangled states and second E5, using the set numerically generated EWs to detect 86.6% of the bound states. The later especially leverages the symmetry analysis to detect additional states. E3 and E4, although less effective than the other criteria, still detect a significant portion (19.1% and 13.5%) of PPT entangled states. Note also that E3 is the criterion that detects all bound entangled states for the family $\mathscr {F}_A$.

**Figure 5:**
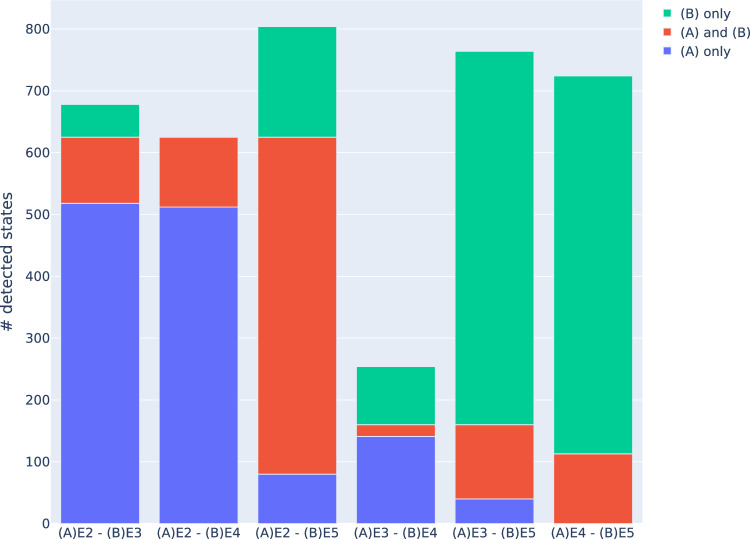
Pairwise comparison of number of exclusively (blue and green) and jointly (red) detected states.

The question arises, whether some of the applied criteria to detect bound entangled states are stronger than others for $E_3$. A criterion (A) is said to stronger than criterion (B), if (A) detects all states detected by (B), as well. Table 4 relates a pair of applied entanglement criteria by comparing the number of exclusively detected bound entangled states to the number of bound entangled states that were detected by both criteria. The results are visualized in Fig. 5.

**Table 4:** Pairwise comparison by criterion of detected bound states.

Criterion (A)	#Detected (A)	Criterion (B)	#Detected (B)	#Detected (A) and (B)
E2	625	E3	160	107
E2	625	E4	113	113
E2	625	E5	724	545
E3	160	E4	113	19
E3	160	E5	724	120
E4	113	E5	724	113

The set of bound entangled states detected by the two most effective criteria, E2 and E5, have a significant intersection, but each criterion also detects states that the other does not. 53 of 160 states (33%) that are detected by E3 are not found by E2, showing that E3 is not really weaker than E2, but seems to detect states of different areas of the geometrical polytope $\mathscr {E}_3$. In this sense, the analytical criteria E2 and E3 complement each other to a certain degree, as they together detect 81% of all detected bound entanglement. A similar, but slightly weaker conclusion can be made for the relation of E3 and E5, where 25% of the states found by E3 are not found by E5. This is in contrast to the results of E4. Here, both E2 and E5 detect all of the states found to be entangled according to E4, too. However, only $17\%$ of the states detected by E4 are also found by E3, showing again a structural difference in the detection capabilities of the criteria E3 and E2. Also note that more than two entanglement criteria are needed to detect all bound entangled states.

In summary, the analytical criterion E2 and the numerically generated E5 are the most effective detectors. Their detected states have a significant intersection, but none is stronger than the other. In principle, all bound entangled states can be detected by E5 with sufficiently many generated EWs. Thus, the states detected by E2 and not by E5 correspond to states in the space between the convex set of separable states and the enclosing hyper-planes of generated linear EWs. E3 detects less states than E2, but has a high share of exclusively detected states. E4 can be considered as weaker than E2 and has a very small overlap with E3, which further supports the hypothesis that E3 has a structural difference compared to the other criteria.

## Discussion

We investigated mixtures of maximally entangled, bipartite Bell states in three dimensions. The states are locally maximally mixed, so all information is in the correlation of the subsystems, while no information about the individual systems is available. Depending on the mixing probabilities, the mixed state can be separable or entangled. Curiously, also bound entangled states, i.e. states that cannot be distilled by LOCC, occur, motivating a detailed analysis of this system to study this exotic form of entanglement.

The investigated system allows a geometric representation in which its states and their properties can be effectively geometrically analyzed. Leveraging these properties, the NP-hard problem of determining whether a state is separable, bound or free entangled can be almost completely solved for Bell diagonal qutrits with new efficient methods. Three main aspects contribute to this result: First, an efficient creation of states to estimate the relative volumes of entanglement classes. Second, the numerical creation of symmetries and their utilization for entanglement classification. Third, the development of independent separability and entanglement detection criteria, which can be applied efficiently to the to be classified states. A new collection of computational methods has been implemented to realize these aspects. It has been shown that the random sampling of states can be used to estimate the relative volumes of entanglement classes without systematic bias and that is has significant advantages compared to using states on a fixed lattice^29^. Further utilizing the generators of the considered symmetries explicitly, all elements of the related group could be generated and applied to improve applied criteria to analyze the entanglement structure. The rapid growth of the number of distinct symmetry transformations with the dimension suggests that our methods can also be applied to higher dimensions despite the exponential growth of the Hilbert space.

Leveraging an efficient parameterization of separable states and the generation of symmetries allowed to use a sufficient criteria to directly detect separable states as well as bound entangled states using numerically generated entanglement witnesses. Several other well known entanglement criteria such as the PPT or realignment criterion have been implemented and applied to a representative sample of the system, allowing to compare their effectiveness.

Two relevant subsets of bipartite states in $\mathscr {M}_d$ for $d=3$ were analyzed. First family $\mathscr {F}_A$, which contains mixtures of three Bell states and the maximally mixed state and whose symmetries allow to show that all bound entangled states can be detected by an analytical witness (E3)^25,32^. More than 99% of the states could be classified including the detection of bound entanglement, but only 0.1% are found to be of that class. The numerical implementation of E3 confirms its effectiveness, since all bound entangled states are detected by this criterion. Interestingly, none of the other criteria detect any bound entanglement for this family. The second analysis and main result of this work is the entanglement classification of the enclosure polytope $\mathscr {E}_3$, which is known to contain all PPT states. Again 10,000 uniformly distributed states were generated as representative sample of the system of which 96.9% could be classified. Only for 3.1% it remains unknown whether the state is separable of bound entangled. Almost half (48.6%) are found to be certainly separable, 40.0% are free entangled and can therefore be distilled by LOCC and at least 8.4% are bound entangled. The developed numerical methods S1 and E5 leveraging the special properties of the investigated system are the most effective criteria to distinguish separable and bound entangled states. However, also general analytical criteria detect significant shares of the bound entangled states, especially the “realignment criterion” E2 is very effective as well. All methods used to detect bound entanglement were investigated in pairwise comparison. Although not being the most effective method in terms of total number of detected states, the nonlinear criteria E3 (“quasi-pure concurrence”), detects a significant amount of states that are not detected by other criteria, especially the linear witness E4 and the collection of linear witnesses E5. This indicates that the states detected by E3 and not by E4 and E5 relate to states close to the nonlinear surface of separable states. It is interesting that E3 is such a strong detector of bound entanglement in $\mathscr {F}_A$, while other criteria like E2, which is a strong detector in $\mathscr {E}_3$, fail for this specific subset.

The developed methods and constructed related objects (i.e. generated entanglement witnesses and extended kernel) can be repeatedly applied to new states of interest in an efficient way. In this sense, the NP-hard problem of entanglement classification is efficiently solved for this system with an accuracy of approximately 94.9%, because any Bell diagonal PPT state can be efficiently classified with high probability. Only for 5.1% of the analyzed PPT states we fail to classify it as separable of bound entangled. Moreover, we can deduce from our studies that least 13.9% of PPT Bell diagonal states are bound entangled while 81.0% are separable. The implemented framework can equivalently be applied to any dimension *d*. It will be interesting to see the results in higher dimensions $d=4$ and $d=5$ and what structural similarities and differences in the classification of entanglement can be identified.

## Supplementary Information

Supplementary Information.

## Data Availability

All analyzed datasets were generated during the current study and are available from the corresponding author on reasonable request.
