# Supplementary material for: Almost complete solution for the NP-hard separability problem of Bell diagonal qutrits
Source: Sci Rep. 2022 Jul 21;12:12472. doi: 10.1038/s41598-022-16225-z (PMC9304426; doi:10.1038/s41598-022-16225-z)
Supplement: Supplementary file 1 — Supplementary Information. [file 41598_2022_16225_MOESM1_ESM.pdf]

Appendix

A1: Entanglement class conserving symmetries

It was shown that the symmetries presented in Ref.[25] for $d = 3$ and in Ref.[24] for general dimension conserve entanglement for states in $\mathcal{M}_d$, i.e. separable states are mapped to separable states and entangled states to entangled states. However, no statement is directly made about the possibility of free entangled states being mapped to bound entangled states and vice versa. That this does not happen and that therefore the symmetries conserve the entanglement class of separable, bound and free states can be seen by the following argument.

Lemma 1. *The entanglement conserving symmetries conserve the entanglement class in $\mathcal{M}_d$.*

Proof. Let $\rho \in \mathcal{M}_d$ and S be the group of all entanglement conserving symmetries. Denote any element $s \in S, s: \mathcal{M}_d \to \mathcal{M}_d$ and the existing inverse by s^{-1}. Denote the set of states with positive partial transposition as PPT and the complementary set as NPT containing free entangled states.The generators of S are represented either by local unitary transformations or the global complex conjugation. Both map PPT onto itself so

$$s: PPT \cap \mathcal{M}_d \to PPT \cap \mathcal{M}_d. \tag{1}$$

Now suppose $s: NPT \cap \mathcal{M}_d \to PPT \cap \mathcal{M}_d$ then $\mathbb{1} = s^{-1}s: NPT \cap \mathcal{M}_d \to PPT \cap \mathcal{M}_d \to PPT \cap \mathcal{M}_d$ is a contradiction. Here the first relation is the assumption and the second follows from (1). So we also have:

$$s: NPT \cap \mathcal{M}_d \to NPT \cap \mathcal{M}_d \tag{2}$$

Denote the set of bound entangled states by $BOUND$. A similar argument shows that bound states are again mapped to bound states: By definition of entanglement conserving symmetries we have

$$S: SEP \cap \mathcal{M}_d \to SEP \cap \mathcal{M}_d. \tag{3}$$

Now suppose $s: BOUND \cap \mathcal{M}_d \to SEP \cap \mathcal{M}_d$ then $\mathbb{1} = s^{-1}s: BOUND \cap \mathcal{M}_d \to SEP \cap \mathcal{M}_d \to SEP \cap \mathcal{M}_d$ is a contradiction. Again the first relations is the assumption and the second from (3). Together with (1) this shows that bound entangled states are mapped to entangled states which are also PPT:

$$S: BOUND \cap \mathcal{M}_d \to BOUND \cap \mathcal{M}_d. \tag{4}$$

(2), (3) and (4) together show the invariance of the classes free entangled, separable and bound entangled under the entanglement conserving symmetries. □

A2: Parameterization of separable states to determine bounds for numeric EWs

An efficient parameterization of the set of separable states is required for entanglement detection with numeric EWs (E5). Using an EW W requires the determination of its upper and lower bound for the set of separable states $\rho_s \in SEP$: $L \leq \mathrm{tr}[\rho_s W] \leq U$. To determine these bounds numerically, a parameterization of separable state is required. Due to the linearity of the trace and the fact that general separable states are defined as convex mixtures of pure states, it suffices to consider pure separable states $\rho_s \in \mathcal{H}_1 \otimes \mathcal{H}_2$ to maximize/minimize $\mathrm{tr}[\rho_s W]$. Any such state can be generated by a local unitary transformation of the state $|00\rangle\langle 00| \equiv |0\rangle\langle 0| \otimes |0\rangle\langle 0|$:

$$\rho_s = \rho_1 \otimes \rho_2 = U_1 |0\rangle\langle 0| U_1^\dagger \otimes U_2 |0\rangle\langle 0| U_2^\dagger = U_1 \otimes U_2 |00\rangle\langle 00| U_1^\dagger \otimes U_2^\dagger \equiv U |00\rangle\langle 00| U^\dagger \tag{5}$$

In Ref.[40] an efficient parameterization of unitaries was proposed that requires only $2(d-1)$ parameters to construct any pure state $\rho_{1/2}$. Consequently, defining a separable, bipartite state ρ_s requires $4(d-1)$ parameters. Naturally, also mixtures of K pure states can be generated accordingly by using $4K(d-1)$ parameters to generate K pure states to be mixed with probabilities $p_1, \ldots, p_K$. The optimization over all separable states to find $\rho_{min/max}$ for a given EW W is carried out for the parameters of the composite parameterization of unitaries according to (5). For $d = 3$, 8 independent parameters need to be optimized in a bounded region. This is done numerically, using the implementation of the "Optim"[43] package of the "LBFGS" algorithm which uses the gradient and an approximation of the Hessian of $\mathrm{tr}[W\rho]$. To minimize the risk of identifying only a local maximum/minimum, the optimization procedures is performed 50 times with random starting points of the algorithm and the overall minimum/maximum is taken for L/U.

A3: Representation for the generators of the considered symmetry group

The details of the construction of (anti-)unitary transformations corresponding to the generators of the considered symmetry groups has been introduced in Ref.[24]. They act as permutations of the Bell basis element $P_{k,l}$ and are applied to any diagonal state in the Bell basis by multiplication of the diagonal elements with the according permutation matrix. Let c be the coordinate vector collecting the elements $c_{k,l},\ k,l = 0,1,2$ of the diagonal density matrix of a state in $\mathscr{M}_3$ represented in the Bell basis $\{P_{k,l}\}$. The symmetries act as permutation on the basis elements $P_{k,l}$ or equivalently on the elements $c_{k,l}$ with the inverse permutation. For a symmetry s acting on the coordinates c with permutation matrix M_s, the coordinates $\tilde{c}$ of the transformed state are then given by $\tilde{c} = M_s \cdot c$. Enumerating the basis elements as $P_{0,0}, P_{1,0}, P_{2,0}, P_{2,1}, \ldots, P_{1,2}, P_{2,2}$, the permutation matrices for the generators are given below (all indices are defined by $\pmod 3$).

Momentum inversion: $m : P_{k,l} \to P_{-k,l}$:

$$M_m = \begin{bmatrix} 1 & 0 & 0 & 0 & 0 & 0 & 0 & 0 & 0 \\ 0 & 0 & 1 & 0 & 0 & 0 & 0 & 0 & 0 \\ 0 & 1 & 0 & 0 & 0 & 0 & 0 & 0 & 0 \\ 0 & 0 & 0 & 1 & 0 & 0 & 0 & 0 & 0 \\ 0 & 0 & 0 & 0 & 0 & 1 & 0 & 0 & 0 \\ 0 & 0 & 0 & 0 & 1 & 0 & 0 & 0 & 0 \\ 0 & 0 & 0 & 0 & 0 & 0 & 1 & 0 & 0 \\ 0 & 0 & 0 & 0 & 0 & 0 & 0 & 0 & 1 \\ 0 & 0 & 0 & 0 & 0 & 0 & 0 & 1 & 0 \end{bmatrix}$$

Quarter rotation: $r : P_{k,l} \to P_{k,-l}$,

$$M_r = \begin{bmatrix} 1 & 0 & 0 & 0 & 0 & 0 & 0 & 0 & 0 \\ 0 & 0 & 0 & 1 & 0 & 0 & 0 & 0 & 0 \\ 0 & 0 & 0 & 0 & 0 & 0 & 1 & 0 & 0 \\ 0 & 0 & 1 & 0 & 0 & 0 & 0 & 0 & 0 \\ 0 & 0 & 0 & 0 & 0 & 1 & 0 & 0 & 0 \\ 0 & 0 & 0 & 0 & 0 & 0 & 0 & 0 & 1 \\ 0 & 1 & 0 & 0 & 0 & 0 & 0 & 0 & 0 \\ 0 & 0 & 0 & 0 & 1 & 0 & 0 & 0 & 0 \\ 0 & 0 & 0 & 0 & 0 & 0 & 0 & 1 & 0 \end{bmatrix}$$

Vertical sheer: $v : P_{k,l} \to P_{k+l,l}$,

$$M_v = \begin{bmatrix} 1 & 0 & 0 & 0 & 0 & 0 & 0 & 0 & 0 \\ 0 & 1 & 0 & 0 & 0 & 0 & 0 & 0 & 0 \\ 0 & 0 & 1 & 0 & 0 & 0 & 0 & 0 & 0 \\ 0 & 0 & 0 & 0 & 0 & 1 & 0 & 0 & 0 \\ 0 & 0 & 0 & 1 & 0 & 0 & 0 & 0 & 0 \\ 0 & 0 & 0 & 0 & 1 & 0 & 0 & 0 & 0 \\ 0 & 0 & 0 & 0 & 0 & 0 & 0 & 1 & 0 \\ 0 & 0 & 0 & 0 & 0 & 0 & 0 & 0 & 1 \\ 0 & 0 & 0 & 0 & 0 & 0 & 1 & 0 & 0 \end{bmatrix}$$

Translation: $t_{p,q} : P_{k,l} \to P_{k+p,l+q}$, e.g.

$$M_{t_{01}} = \begin{bmatrix} 0 & 0 & 0 & 0 & 0 & 0 & 1 & 0 & 0 \\ 0 & 0 & 0 & 0 & 0 & 0 & 0 & 1 & 0 \\ 0 & 0 & 0 & 0 & 0 & 0 & 0 & 0 & 1 \\ 1 & 0 & 0 & 0 & 0 & 0 & 0 & 0 & 0 \\ 0 & 1 & 0 & 0 & 0 & 0 & 0 & 0 & 0 \\ 0 & 0 & 1 & 0 & 0 & 0 & 0 & 0 & 0 \\ 0 & 0 & 0 & 1 & 0 & 0 & 0 & 0 & 0 \\ 0 & 0 & 0 & 0 & 1 & 0 & 0 & 0 & 0 \\ 0 & 0 & 0 & 0 & 0 & 1 & 0 & 0 & 0 \end{bmatrix}$$

References

1. Nielsen, M. A. & Chuang, I. L. *Quantum Computation and Quantum Information* (Cambridge University Press, 2000).
2. Cozzolino, D., Da Lio, B., Bacco, D. & Oxenløwe, L. K. High-dimensional quantum communication: Benefits, progress, and future challenges. *Advanced Quantum Technologies* **2**, 1900038 (2019). URL https://doi.org/10.1002/qute.201900038.
3. Wang, Y., Hu, Z., Sanders, B. C. & Kais, S. Qudits and high-dimensional quantum computing. *Frontiers in Physics* **8**, 479 (2020). URL https://www.frontiersin.org/article/10.3389/fphy.2020.589504.
4. Moskal, P. *et al.* Time resolution of the plastic scintillator strips with matrix photomultiplier readout for j-PET tomograph. *Physics in Medicine and Biology* **61**, 2025–2047 (2016). URL https://doi.org/10.1088/0031-9155/61/5/2025.
5. Moskal, P. & Stepien, E. Prospects and clinical perspectives of total-body pet imaging using plastic scintillators. *PET Clinics* **15** (2020). URL https://doi.org/10.1016/j.cpet.2020.06.009.
6. Hiesmayr, B. C. & Moskal, P. Genuine multipartite entanglement in the 3-photon decay of positronium. *Scientific Reports* **7**, 15349 (2017). URL https://doi.org/10.1038/s41598-017-15356-y.
7. Hiesmayr, B. C. & Moskal, P. Witnessing entanglement in compton scattering processes via mutually unbiased bases. *Scientific Reports* **9**, 8166 (2019). URL https://doi.org/10.1038/s41598-019-44570-z.
8. Horodecki, M., Horodecki, P. & Horodecki, R. Mixed-state entanglement and distillation: Is there a "bound" entanglement in nature? *Phys. Rev. Lett.* **80**, 5239–5242 (1998). URL https://link.aps.org/doi/10.1103/PhysRevLett.80.5239.
9. Bej, P. & Halder, S. Unextendible product bases, bound entangled states, and the range criterion. *Physics Letters A* **386**, 126992 (2021). URL https://www.sciencedirect.com/science/article/pii/S0375960120308598.
10. Lockhart, J., Gühne, O. & Severini, S. Entanglement properties of quantum grid states. *Phys. Rev. A* **97**, 062340 (2018). URL https://link.aps.org/doi/10.1103/PhysRevA.97.062340.
11. Bruß, D. & Peres, A. Construction of quantum states with bound entanglement. *Phys. Rev. A* **61**, 030301 (2000). URL https://link.aps.org/doi/10.1103/PhysRevA.61.030301.
12. Slater, P. B. Jagged islands of bound entanglement and witness-parameterized probabilities. *arXiv: Quantum Physics* (2019). URL https://doi.org/10.48550/arXiv.1905.09228.
13. Choi, M.-D. Some assorted inequalities for positive linear maps on c*-algebras. *Journal of Operator Theory* **4** (1980).
14. Chruściński, D. & Sarbicki, G. Entanglement witnesses: construction, analysis and classification. *J. Phys. A: Math. Theor.* **47**, 483001 (2014). URL https://doi.org/10.1088/1751-8113/47/48/483001.
15. Kalev, A. & Bae, J. Optimal approximate transpose map via quantum designs and its applications to entanglement detection. *Phys. Rev. A* **87**, 062314 (2013). URL https://link.aps.org/doi/10.1103/PhysRevA.87.062314.
16. Bae, J. Designing quantum information processing via structural physical approximation. *Reports on Progress in Physics* **80**, 104001 (2017). URL https://doi.org/10.1088/1361-6633/aa7d45.
17. Korbicz, J. K., Almeida, M. L., Bae, J., Lewenstein, M. & Acín, A. Structural approximations to positive maps and entanglement-breaking channels. *Phys. Rev. A* **78**, 062105 (2008). URL https://link.aps.org/doi/10.1103/PhysRevA.78.062105.
18. Huber, M., Mintert, F., Gabriel, A. & Hiesmayr, B. C. Detection of high-dimensional genuine multipartite entanglement of mixed states. *Phys. Rev. Lett.* **104**, 210501 (2010). URL https://link.aps.org/doi/10.1103/PhysRevLett.104.210501.
19. Augusiak, R., Bae, J., Tura Brugués, J. & Lewenstein, M. Checking the optimality of entanglement witnesses: An application to structural physical approximations. *J. Phys. A: Math. Theor.* **47** (2014). URL https://iopscience.iop.org/article/10.1088/1751-8113/47/6/065301.
20. Hiesmayr, B. C. & Löffler, W. Complementarity reveals bound entanglement of two twisted photons. *New J. Phys* **15**, 083036 (2013). URL https://doi.org/10.1088/1367-2630/15/8/083036.
21. Peres, A. Separability criterion for density matrices. *Phys. Rev. Lett.* **77**, 1413–1415 (1996). URL https://link.aps.org/doi/10.1103/PhysRevLett.77.1413.

22. Horodecki, M., Horodecki, P. & Horodecki, R. Separability of mixed states: necessary and sufficient conditions. *Physics Letters A* **223**, 1–8 (1996). URL https://www.sciencedirect.com/science/article/pii/S0375960196007062.

23. Gurvits, L. Classical deterministic complexity of edmonds' problem and quantum entanglement. In *Proceedings of the Thirty-Fifth Annual ACM Symposium on Theory of Computing*, STOC '03, 10–19 (Association for Computing Machinery, New York, NY, USA, 2003). URL https://doi.org/10.1145/780542.780545.

24. Baumgartner, B., Hiesmayr, B. C. & Narnhofer, H. A special simplex in the state space for entangled qudits. *J. Phys. A: Math. Theor.* **40**, 7919 (2007). URL https://doi.org/10.1088/1751-8113/40/28/S03.

25. Baumgartner, B., Hiesmayr, B. C. & Narnhofer, H. State space for two qutrits has a phase space structure in its core. *Phys. Rev. A* **74**, 032327 (2006). URL https://link.aps.org/doi/10.1103/PhysRevA.74.032327.

26. Baumgartner, B., Hiesmayr, B. C. & Narnhofer, H. The geometry of bipartite qutrits including bound entanglement. *Physics Letters A* **372**, 2190–2195 (2008). URL https://www.sciencedirect.com/science/article/pii/S0375960107016507.

27. Życzkowski, K., Horodecki, P., Sanpera, A. & Lewenstein, M. Volume of the set of separable states. *Phys. Rev. A* **58**, 883–892 (1998). URL https://link.aps.org/doi/10.1103/PhysRevA.58.883.

28. Zyczkowski, K. Volume of the set of separable states. ii. *Phys. Rev. A* **60**, 3496–3507 (1999). URL https://link.aps.org/doi/10.1103/PhysRevA.60.3496.

29. Hiesmayr, B. C. Free versus bound entanglement, a np-hard problem tackled by machine learning. *Scientific Reports* **11**, 19739 (2021). URL https://doi.org/10.1038/s41598-021-98523-6.

30. Bennett, C. H. *et al.* Teleporting an unknown quantum state via dual classical and einstein-podolsky-rosen channels. *Phys. Rev. Lett.* **70**, 1895–1899 (1993). URL https://link.aps.org/doi/10.1103/PhysRevLett.70.1895.

31. Hill, S. A. & Wootters, W. K. Entanglement of a pair of quantum bits. *Phys. Rev. Lett.* **78**, 5022–5025 (1997). URL https://link.aps.org/doi/10.1103/PhysRevLett.78.5022.

32. Bae, J. *et al.* Detection and typicality of bound entangled states. *Phys. Rev. A* **80**, 022317 (2009). URL https://link.aps.org/doi/10.1103/PhysRevA.80.022317.

33. Chen, K. & Wu, L.-A. A matrix realignment method for recognizing entanglement. *Quantum Information and Computation* **3** (2002). URL https://doi.org/10.48550/arXiv.quant-ph/0205017.

34. Wootters, W. K. Entanglement of formation of an arbitrary state of two qubits. *Phys. Rev. Lett.* **80**, 2245–2248 (1998). URL https://link.aps.org/doi/10.1103/PhysRevLett.80.2245.

35. Spengler, C., Huber, M., Brierley, S., Adaktylos, T. & Hiesmayr, B. C. Entanglement detection via mutually unbiased bases. *Phys. Rev. A* **86**, 022311 (2012). URL https://link.aps.org/doi/10.1103/PhysRevA.86.022311.

36. Wootters, W. K. & Fields, B. D. Optimal state-determination by mutually unbiased measurements. *Annals of Physics* **191**, 363–381 (1989). URL https://www.sciencedirect.com/science/article/pii/0003491689903229.

37. Bandyopadhyay, S., Boykin, P. O., Roychowdhury, V. P. & Vatan, F. A new proof for the existence of mutually unbiased bases. *Algorithmica* **34**, 512–528 (2002). URL https://doi.org/10.1007/s00453-002-0980-7.

38. Terhal, B. M. Bell inequalities and the separability criterion. *Physics Letters A* **271**, 319–326 (2000). URL https://www.sciencedirect.com/science/article/pii/S0375960100004011.

39. Bae, J., Chruściński, D. & Hiesmayr, B. C. Mirrored entanglement witnesses. *npj Quantum Information* **6** (2020). URL https://doi.org/10.1038/s41534-020-0242-z.

40. Spengler, C., Huber, M. & Hiesmayr, B. C. A composite parameterization of unitary groups, density matrices and subspaces. *J. Phys. A: Math. Theor* **43**, 385306 (2010). URL https://doi.org/10.1088/1751-8113/43/38/385306.

41. Forets, M. & Schilling, C. Lazysets.jl: Scalable symbolic-numeric set computations*. *Proceedings of the JuliaCon Conferences* **1**, 97 (2021). URL https://doi.org/10.21105/jcon.00097.

42. Chruściński, D. & Pittenger, A. O. Generalized circulant densities and a sufficient condition for separability. *J. Phys. A: Math. Theor.* **41**, 385301 (2008). URL https://doi.org/10.1088/1751-8113/41/38/385301.

43. Mogensen, P. K. & Riseth, A. N. Optim: A mathematical optimization package for Julia. *Journal of Open Source Software* **3**, 615 (2018). URL https://doi.org/10.21105/joss.00615.
